# Induction of strain-transcendent antibodies to placental-type isolates with VAR2CSA DBL3 or DBL5 recombinant proteins

**DOI:** 10.1186/1475-2875-10-36

**Published:** 2011-02-11

**Authors:** Marion Avril, Megan M Cartwright, Marianne J Hathaway, Joseph D Smith

**Affiliations:** 1 Seattle Biomedical Research Institute, 307 Westlake Ave N, Suite 500, Seattle Washington, 98109-5219, USA; 2 Department of Global Health, University of Washington, Seattle, WA, 98195, USA

## Abstract

**Background:**

Pregnancy associated malaria is a severe clinical syndrome associated with sequestration of *Plasmodium falciparum*-infected erythrocytes in the placenta. Placental binding is mediated by VAR2CSA, which adheres to chondroitin sulphate A (CSA). VAR2CSA is a large and polymorphic protein that has six Duffy binding-like (DBL) domains. There is still limited understanding as to how effective individual VAR2CSA domains are at generating inhibitory antibodies or the number of domain variants needed for universal vaccine coverage.

**Methods:**

To investigate the immunogenic properties of single domain VAR2CSA recombinant proteins, rats or rabbits were immunized with five of the six VAR2CSA domains produced in *Pichia pastoris*. Immune plasma was analysed against a geographically diverse panel of CSA-binding lab lines to assess antibody breadth and inhibitory activity.

**Results:**

Of the five domains, DBL3, and to a lesser extent DBL5, induced antibodies that cross-reacted on five diverse CSA-binding parasite lines by flow cytometry. By comparison, anti-DBL6 antibodies were highly strain-specific and anti-DBL1 and anti-DBL4 antibodies were poorly reactive by flow cytometry. From this series of recombinant proteins, adhesion-blocking activity was restricted to a single rat immunized against a DBL4 recombinant protein.

**Conclusions:**

Single domain VAR2CSA recombinant proteins produced in *P. pastoris *had limited efficacy in eliciting adhesion blocking antibody responses, but VAR2CSA DBL3 and DBL5 domains contain strain-transcendent epitopes that can be targeted by vaccination and may have application for vaccine development.

## Background

Despite important advances, the burden of malaria remains very high, with more than 2.4 billion people at risk of malaria. Approximately 50 million women of child-bearing age are exposed to this risk of malaria every year [1,2]. Pregnancy associated malaria is a major cause of poor mother and child health and leads to maternal anemia, prematurity, low birth weight and increased infant morbidity and mortality [3]. This syndrome is associated with *Plasmodium falciparum *infected erythrocytes (IEs) that selectively sequester in the placenta via binding chondroitin sulfate A (CSA) [4,5]. Women become resistant to pregnancy malaria over the course of multiple pregnancies as they acquire antibodies that recognize placental isolates from geographically diverse regions [6-8], suggesting it may be feasible to develop a vaccine. Antibodies are thought to contribute to protection by blocking adhesion of IEs to CSA and by opsonizing IEs for phagocytosis [6,7,9-11].

Placental binding is associated with an unusually conserved *var *gene, VAR2CSA, which is transcriptionally up-regulated in CSA binding parasites and expressed at the surface of placental IEs [12,13]. Genetic disruption of *var2CSA *largely abolishes CSA-binding [14-16] suggesting it is the major *var *encoded product associated with placental sequestration. These findings support the development of a VAR2CSA-based vaccine against placental malaria. However, sequence analysis has revealed diversity among global isolates [17-19], which poses challenges for developing a universal vaccine.

The VAR2CSA extracellular region contains six Duffy binding-like (DBL) adhesion domains [13]. Several individual DBL domains (DBL2, DBL3, and DBL6) have been reported to bind to CSA and a co-crystal has been solved for VAR2CSA DBL3-CSA [20,21]. However, it has been questioned whether binding interactions of single domains are physiologically relevant because many randomly expressed DBL domains from other members of the *var *gene family also bind to CSA [22,23]. Furthermore, the full-length VAR2CSA protein binds with much greater specificity and affinity than individual domains [24,25]. Thus, it remains unclear whether VAR2CSA has one or multiple CSA-interaction sites, and binding site(s) in the full-length DBL1-6 recombinant protein remains uncharacterized.

Immunization of animals with single domain VAR2CSA recombinant proteins produced in *Baculovirus *[26], *Escherichia coli *[27,28] and *Pichia pastoris *[29,30] demonstrate that it is possible to generate antibodies reactive with native VAR2CSA at the IE surface. However, there has been limited investigation into the breath of antibody reactivity and it remains difficult to induce inhibitory antibodies. To date, few DBL recombinant antigens have induced anti-adhesive antibodies [28,31,32], except for an IT4-DBL4-VAR2CSA recombinant protein produced in *Baculovirus *[31], and a refolded IT4-DBL5 recombinant protein produced in *Escherichia coli *[33]. However, adhesion-blocking responses have been variable between different DBL4 and DBL5 antigen preparations and sensitive to construct boundaries [32,34]. The best DBL4 recombinant protein induced a broad adhesion blocking response to a range of different placental isolates [34], but not all parasites isolates were inhibited [34], and inhibitory antibodies were only observed against one of four different DBL4 alleles tested [32]. Thus, this approach has potential but more work is needed to optimize single domain immunogens for pregnancy malaria vaccine development.

In this study, the immunogenicity of single domain VAR2CSA recombinant proteins was investigated by immunizing rats and rabbits with *P. pastoris *DBL engineered immunogens. Immune sera were examined against both the homologous parasite line and a diverse panel of CSA binding parasite lines to identify DBL domains that elicited a broader antibody response and to define extracellular regions that contained adhesion-blocking epitopes.

## Methods

### Recombinant protein expression in *Pichia pastoris*

**Figure 1:**
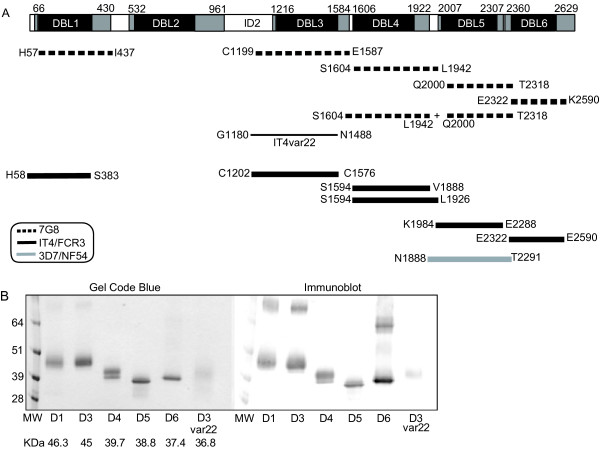
**Expression of VAR2CSA-DBL recombinant proteins in *P. pastoris***. A) Protein schematic of VAR2CSA. The original DBL domain boundaries are indicated by black rectangles. Revised domain boundaries as described [35] are indicated in grey and numbered for the 7G8-VAR2CSA allele. The first and last amino acids of constructs are indicated below the schematic. The thin line refers to the non-*var2csa *encoded protein IT4var22 DBL3. B) 1 μg of His-tagged recombinant proteins were analysed under non-reducing conditions in a 4-20% SDS-PAGE gel and stained by Gel Code Blue Reagent or detected by immunoblot via anti-His tag antibodies. New 7G8-VAR2CSA and IT4var22 DBL3 recombinant proteins generated for this study are shown. Other recombinant proteins were described previously [35].

Cloning and production of 7G8-VAR2CSA recombinant proteins was done in *Pichia pastoris *as previously described [29,35]. Construct boundaries are indicated in Figure 1. Recombinant proteins were analysed in 4-20% SDS-PAGE gels under non-reducing conditions. Gels were stained with Gel Code Blue Reagent or transferred to a nitrocellulose membrane and detected by Western Blot using anti-His tag antibodies (Invitrogen). The identity of recombinant proteins was confirmed by mass spectrometry analyses. Purified proteins were stored at -80°C in 1× phosphate buffered saline.

### Animal immunization

Immunizations were performed at R&R Rabbitry (Washington, USA) according to animal immunization guidelines. Immunizations were approved by the Institute Animal and Care Use Committee at Seattle Biomedical Research Institute and at R&R Rabbitry (Vendor's PHS assurance # A3982-01 and USDA registration 91-R-0038). In brief, three rabbits or three rats per group received the recombinant protein in complete Freund's adjuvant for the first immunization and were boosted with antigen in incomplete Freund's adjuvant. Each animal received the antigen subcutaneously every three weeks, for a total of four times for rats and four to five times for rabbits (Table 1). All injections followed the same protocol and the same dose was administrated for priming and boosts. Rabbits received between 50 to 100 μg of recombinant protein while rats received between 20 to 40 μg. Pre-immune and immune plasma were heat-inactivated for 45 min at 57°C and stored at -20°C. Prior to serological assays, plasma were preabsorbed twice on uninfected O^+ ^erythrocytes.

**Table 1:** Immunization protocol

Immunogen VAR2CSA		Animal	**Number of injections **^**1**^	Vaccination doses (μg)	
				**Prime **^**2**^	**Boost **^**3**^

DBL1	7G8	3 rabbits ^4^	5	100	100
		3 rats	4	20	20
	IT4	2 rabbits ^5^	6	500	250

DBL3	7G8	3 rats	4	20	20
	IT4	3 rabbits	4	50	50

DBL4 short	IT4	3 rats	4	20	20
		2 rabbits ^5^	6	500	250

DBL4 long	7G8	3 rats	4	20	20
	IT4	3 rats	4	20	20

DBL4+DBL5	7G8	3 rats	4	20+20	20+20

DBL5	7G8	3 rabbits ^4^	5	100	100
		3 rats	4	20	20
	IT4	3 rabbits ^4^	5	25	25
	3D7	3 rabbits ^4^	5	50	50
		3 rats	4	20	20

DBL6	7G8	3 rats	4	20	20
	IT4	2 rabbits ^5^	6	500	250
		3 rats	4	20	20

Immunogen control var22					
DBL3	IT4	3 rabbits ^4^	5	50	50
		3 rats	4	20	20

1. Animals were immunized subcutaneously

2. Recombinant protein in Complete Freund's adjuvant

3. Recombinant protein in Incomplete Freund's adjuvant

4. As previously published [30]

5. As previously published [29]

### Parasites lines

*Plasmodium falciparum *parasites were grown in O^+ ^erythrocytes and 5% human plasma. CSA binding laboratory lines, IT4/FCR3-CSA (origin ambiguous) [36], 7G8-CSA (South America) [29], HB3-CSA allele A and HB3-CSA allele B (Central America) [29], Pf2004-CSA (West Africa) [37,38] and Pf2006-CSA (West Africa) [37,38], were maintained by periodic selection on CSA. For non-CSA binding controls, two CD36-binding parasite lines were employed that are isogenic to IT4/FCR3. A4ultra expresses *IT4var14 *and ItG-ICAM-1 expresses *IT4var16*. Genotyping of parasites was done with MSP1/MSP2 primers according to published approaches [39]. At the time of antibody assays, RNA was collected and *var2CSA *gene transcription was assessed by qRT-PCR using universal primers against the DBL4 domain, as described previously [29].

### Flow cytometry assay on infected erythrocytes

Mature-stage IEs were grown in O^+ ^blood and incubated with rat or rabbit plasma that had been preabsorbed twice on uninfected O^+ ^erythrocytes. For each assay, 10 million erythrocytes at between 5-8% trophozoites were incubated with a 1/20 dilution of rat plasma or a 1/25 dilution of rabbit plasma. Bound antibodies were detected by adding Alexafluor 488 conjugated goat anti-rat IgG (A-11006, Molecular Probes, 1/500) or Alexafluor 488 conjugated goat anti-rabbit IgG (A-11034, Molecular Probes, 1/500). Samples were analysed in an LSRII (Becton Dickinson) and analysed using FLOWJO 8.1 software (Tree Star Inc). Binding is presented as the mean of the adjusted geometric mean of fluorescence intensity (MFI) for plasma run in duplicate. The adjusted MFI = (IE_i_-UE_i_) - (IE_p_-UE_p_) where IE_i _= MFI of infected erythrocytes following incubation in immune plasma, UE_i _= MFI of uninfected erythrocytes following incubation in immune plasma, IE_p _= MFI of infected erythrocytes following incubation in preimmune plasma, UE_p _= MFI of uninfected erythrocytes following incubation in preimmune plasma.

### Infected erythrocyte binding and antibody binding inhibition assays

Infected erythrocyte binding was performed on CSA-coated bacterial petri dishes as previously described [29]. In brief, infected erythrocytes were tested for binding to 10 μl spots of 0.05 mg/ml bovine CSA (Fluka Biochemika) or 0.05 mg/ml rCD36-Fc (R&D, USA). Binding assays were performed with 10 μl of 1×10^7 ^IEs/ml enriched to 25% to 50% parasitaemia via pork gelatin flotation [40]. Rabbit or rat polyclonal plasma were tested individually or as a pool of plasma at 1/10 final dilution. In antibody binding inhibition assays, IEs were preincubated with immune plasma for 30 minutes before IEs were added to the CSA spots. Binding assays were performed in the presence of immune plasma. The percentage of binding inhibition was calculated relative to a control anti-IT4var22 DBL3 immune plasma.

### ELISA assay

To determine an ELISA titer, rat and rabbit immune plasma were tested against their corresponding *P. pastoris *DBL recombinant protein. Assays were performed using the rabbit (#80160) or rat (#80155) serum antibody detection ELISA kit (Alpha Diagnostic International). Briefly, 96-well ELISA plates (Nunc) were coated with 200 ng recombinant DBL protein and incubated at 4°C overnight. Plates were blocked with a milk-based blocking buffer provided with the kit for 2 hr at room temperature and then serial diluted plasma from 1/500 to 1/512000 was added to antigen-coated wells in duplicate for 1 hr at room temperature. After washes with wash buffer, plates were incubated with horseradish peroxidase-conjugated, goat anti-rabbit IgG or anti-rat IgG diluted 1/2000 for 1 hr, washed for 30 min, and then exposed to Ready-to-Use tetramethylbenzidine substrate for 15 min. Absorbance was read at 450 nm using a microplate reader (Molecular Devices) and analysed by SOFTmaxPRO version 5. Data were graphed with a 4-pt fit curve and antibody titer calculated at 0.1 OD.

## Results

### Production of DBL recombinant proteins in *Pichia pastoris*

To produce single domain VAR2CSA recombinant proteins, constructs were secreted from the methylotrophic yeast *P. pastoris*. Five of six DBL domains (DBL1 and DBLs 3 to 6) were produced from the 7G8-VAR2CSA allele (Figure 1). Yields ranged from 1 mg/L for DBL1 and DBL5, 2 and 3 mg/L for DBL4 and DBL3, and over 10 mg/L for DBL6 domain under routine shaker flask conditions (Figure 1). In contrast, the DBL2 domain could not be produced from 7G8-VAR2CSA, or from two other VAR2CSA alleles previously (IT4/FCR3 or 3D7) [35], despite repeated attempts with different construct boundaries. As a negative control protein for immunization, IT4var22 DBL3 was produced in the same system (Figure 1). IT4var22 DBL3 was expressed at a relatively weak level (< 0.5 mg/L). Under non-reducing conditions, most of the proteins ran at the expected size of monomers (Figure 1). However, a portion of DBL1, DBL3 and DBL6 recombinant proteins migrated at a larger molecular size, possibly due to dimerization, and remained even after boiling and reducing conditions. Overall, five of the six 7G8-VAR2CSA DBL domains could be produced as single domain recombinant proteins from *P. pastoris*.

### VAR2CSA recombinant proteins produced in *P. pastoris *displayed differential ability to elicit surface-reactive antibodies to the homologous parasite

**Figure 2:**
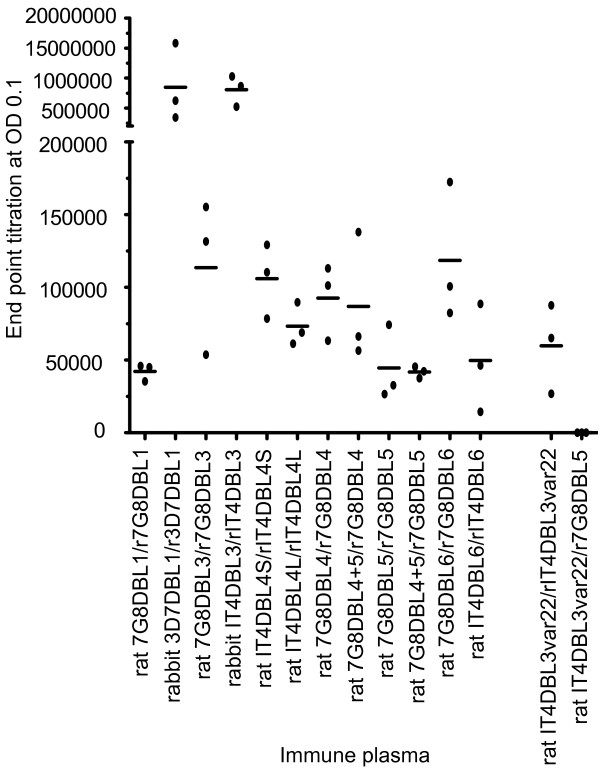
**Endpoint titers of anti-VAR2CSA plasma as determined by ELISA**. The endpoint titers of anti-VAR2CSA plasma at OD 0.1 is shown. Individual animals are indicated by dots. Mean values are indicated by bars. Rat and rabbit immune plasma were tested against their corresponding *P. pastoris *DBL recombinant protein at 200 ng (e.g. immune plasma/recombinant protein).

To investigate the immunogenic properties of single domain proteins, rats were immunized with the five 7G8-VAR2CSA domains or IT4var22 DBL3 control protein (Table 1). In addition, one group of rats received a combination of equal amount of 7G8 DBL4 and 7G8 DBL5 proteins (denoted 7G8 DBL4+5) because these domains have been found to induce adhesion blocking antibody responses in some instances [31,33]. By ELISA, the end point titer of most rat immune plasma ranged between 1 × 10^4 ^and 2 × 10^5 ^against the corresponding VAR2CSA immunogen (Figure 2). The control plasma anti-IT4var22 DBL3 reacted well against its homologous protein IT4var22 DBL3 but not against VAR2CSA *Pichia *7G8 DBL5 recombinant protein.

**Figure 3:**
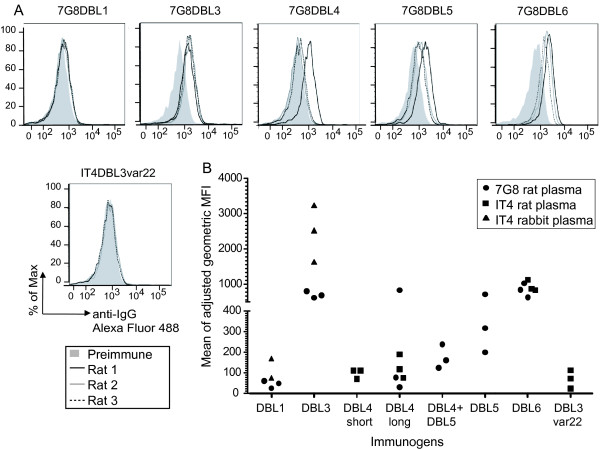
**Cross-reactivity of anti-VAR2CSA on a panel of CSA-binding parasites**. A) Flow cytometry analysis of rat anti-VAR2CSA plasma on the homologous 7G8-CSA parasite line. Preimmune plasma is shown in grey, black line for rat #1, grey for rat #2, and dashed line for rat #3. The reactivity of protein control, anti-IT4var22 DBL3 plasma is shown as well. B) Summary of homologous surface reactivity against 7G8-CSA and IT4-CSA parasite lines. Each dot represents the mean adjusted MFI from duplicate experiments observed for each animal.

**Figure 4:**
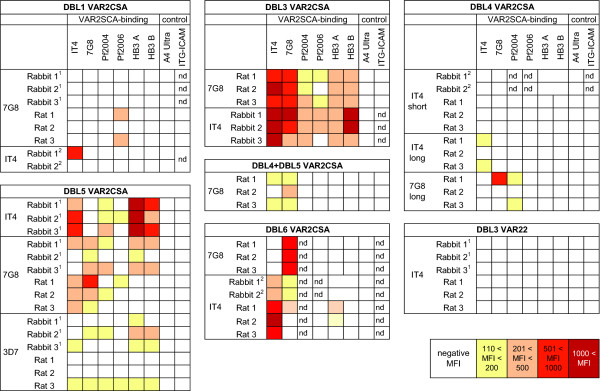
**Summary of the cross reactivity observed per DBL domain across parasite panel**. The top row in each box shows the DBL domain used for immunization. On the left is listed the VAR2CSA allelic variant and animal species immunized. Mean fluorescence intensities in flow cytometry are shown as a heat map from yellow (lower) to red (higher). Rat plasma were analysed at 1/20 dilution and rabbit plasma at 1/25 dilution. Previously published observations are indicated with superscripts: ^1^[30] and ^2^[29]. All plasma were tested against the individual HB3A and HB3B CSA-binding parasite lines except the previously published rabbit anti-IT4 DBL5 and anti-IT4 DBL6 were examined against a mixed HB3-CSA parasite line.

To measure surface reactive antibodies, immune plasma were first examined by flow cytometry against the homologous parasite line. As expected, the control anti-IT4var22 DBL3 plasma did not react with 7G8-CSA infected erythrocytes (Figure 3). Of the five VAR2CSA domains, DBL3, DBL5, and DBL6 recombinant proteins induced the strongest antibody reactivity against the homologous 7G8-CSA parasite (Figure 3), and did not cross-react against two different CD36-binding negative control parasite lines (Figure 4). In contrast, 7G8-DBL1 recombinant protein did not induce good surface reactive antibodies against 7G8-CSA IEs in any of three rats and only one of three rats immunized with 7G8-DBL4 (rat# 1) was reactive with 7G8-CSA (Figure 3). Lastly, the equal mixture of 7G8-DBL4 and 7G8-DBL5 proteins did not produced a greater surface reactivity against the homologous parasite than the DBL4 or DBL5 antigen alone (Figure 3B). Thus, the five proteins differed in their ability to induce surface reactive antibodies.

For comparison, rats or rabbits were immunized with recombinant proteins from a second VAR2CSA allele, IT4-VAR2CSA (Figure 1). In this case, two different versions of IT4-VAR2CSA DBL4 were employed (Figure 1), consisting of a "longer" version (D4L; S1594-L1926) containing the predicted C-terminal cysteine and a "short" version (D4S; S1594-V1888) lacking the final two predicted cysteine residues. Similar to the 7G8 recombinant proteins, antibodies to the DBL3 and DBL6 recombinant proteins were strongly reactive with the homologous parasites, but the DBL1, DBL4S, and DBL4L recombinant proteins did not induce significant reactivity against the homologous parasite line (Figure 3B). Furthermore, rabbits immunized with the IT4-DBL5 domain produced strong antibody responses to the homologous parasite line [30]. Thus, in two different instances, DBL3, DBL5, and DBL6 recombinant proteins produced in *P. pastoris *were superior to DBL1 and DBL4 in eliciting antibodies against the homologous parasite line.

### VAR2CSA DBL3 and DBL5 recombinant proteins induce cross-reactive antibodies to diverse CSA binding parasite lines

To identify whether any of the VAR2CSA domains induced cross-reactive antibodies, immune sera were tested against a panel of five CSA-binding lines from diverse geographic regions and two CD36 binding control parasite lines. As observed for homologous parasite, DBL1 and DBL4 recombinant proteins generally induced weak or no antibody responses against heterologous CSA binding lines (Figure 4). In addition, anti-DBL6 plasma made against either an IT4-DBL6 or a 7G8-DBL6 recombinant protein did not cross-react on heterologous CSA binding parasite lines (Figure 4), possibly because DBL6 is the most polymorphic extracellular domain [18]. In contrast, anti-DBL3 and anti-DBL5 plasma from both rats and rabbits cross-reacted on diverse CSA-binding parasite lines (Figure 4). The extent of antibody cross-reactivity differed between parasite lines, and DBL3 immunogens induced greater breadth than DBL5. Lastly, an equal mixture of 7G8-DBL4 and 7G8-DBL5 proteins did not produce greater surface reactivity with the 7G8-CSA parasite line than DBL4 or DBL5 alone (Figure 4). Previous sequence comparisons showed that VAR2CSA DBL3, DBL4, and DBL5 domains are slightly more conserved than other extracellular domains (82-88% amino acid identity versus 60-80% for other domains) [18]. Within the parasite panel, DBL3 sequences averaged 87% amino acid identity (range 81-91%) and DBL5 sequences had 86% amino acid identity (range 83-99%). Thus, despite the presence of sequence polymorphisms, VAR2CSA DBL3 and DBL5 domains contain cross-reactive epitope(s) that are widely geographically distributed in different CSA-binding parasite lines. In contrast, such broad cross-reactivity was never observed after immunizing with a DBL1, DBL4 or DBL6 recombinant protein in rats or rabbits.

### Individual VAR2CSA DBL domains were ineffective in generating adhesion blocking antibodies

**Figure 5:**
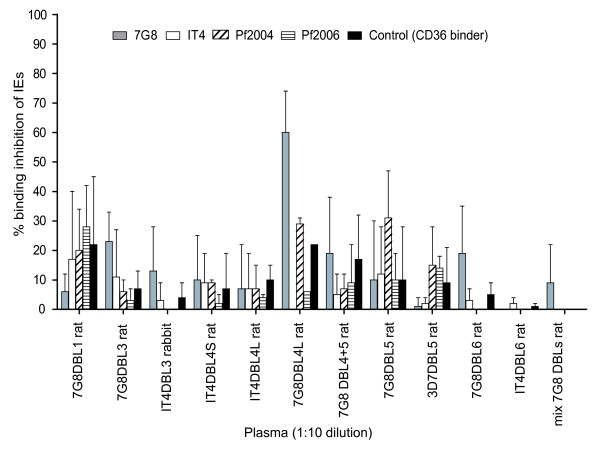
**Binding inhibition activity of anti-VAR2CSA plasma**. Binding inhibitory activity of anti-VAR2CSA plasma were tested in an *in vitro *binding assay with four different CSA-binding parasite lines and a CD36 binding control parasite line. The binding inhibitory activity was tested in a CSA-binding assay for the CSA-binding parasites and in a CD36 binding assay for the CD36 binding parasite. All anti-VAR2CSA plasma were tested as a pool of the three rats in each group, except rat #1 anti-DBL4 plasma was tested on its own because the other two rat plasma did not react by flow cytometry. A mixture of plasma against five 7G8-DBL domains (DBL1, DBL3, DBL4, DBL5, and DBL6) was also tested against the homologous 7G8-CSA parasite line. The extent of binding inhibition was expressed relative to the pool of rat immune anti-IT4var22 DBL3. The mean and range of binding inhibition from duplicate experiments is shown.

To investigate if DBL recombinant proteins produced in *P. pastoris *induced antibodies that could inhibit infected erythrocyte binding to CSA, an in vitro binding inhibition assay was performed using 4 CSA-binding lines (7G8-CSA, IT4-CSA, Pf2004-CSA, Pf2006-CSA). For these assays, pooled plasma was used, except for rat anti-DBL4. In this case, pooled anti-DBL4 plasma was compared to the single rat #1 that was positive by flow cytometry (Figure 3). Binding inhibition was analysed relative to rats immunized with a non-CSA-binding control protein (pooled rat anti-var22 DBL3 plasma). The corresponding pooled preimmune plasma were tested as well but were negative. As shown in Figure 5, the only plasma that exhibited modest inhibition activity was rat #1 anti-DBL4 (60 ± 14% inhibition of 7G8-CSA). However, activity was largely restricted to the homologous parasite line (Figure 5), and binding inhibition was reduced to less than 20% after pooling the three rat's anti-DBL4 plasma. In addition, anti-DBL5 and anti-DBL6 plasma gave minimal inhibition (< 30%) against one of the four CSA-binding parasite lines. Most other plasma had little to no inhibitory activity (≤ 20%) against both homologous and heterologous parasite lines. This low level is considered non-significant because it does not correlate with antibody reactivity against the same parasite lines by flow cytometry (Figure 4) and was similar to the negative control CD36 binding parasite line to CD36 (Figure 5). Furthermore, a combination of plasma to 7G8 DBL1, DBL3, DBL4, DBL5, and DBL6 had less than 10% inhibitory activity on the homologous 7G8-CSA parasite line (Figure 5), similar to the negative control anti-IT4 var22 DBL3 plasma. Therefore, most single domain immunogens produced in *P. pastoris *were ineffective in inducing inhibitory antibodies, except for a single rat immunized with a DBL4 recombinant protein.

## Discussion

The development of a pregnancy malaria vaccine will require the identification of immunogen(s) that can induce broad reactivity to diverse placental isolates. In malaria endemic regions, pregnant women are exposed to many different placental genotypes/VAR2CSA alleles in a single pregnancy [41-43]. Although this cumulative exposure eventually leads to immunity [3], it may be difficult to replicate in a vaccine with mixtures of VAR2CSA recombinant proteins. It is not yet clear whether the extensive breadth of acquired antibodies represents a few highly conserved epitopes or is an accumulation of many different antibody specificities. In addition, the epitopes targeted by adhesion-blocking antibodies are not yet defined. Thus, a pregnancy malaria vaccine may need to induce a response that is qualitatively or quantitatively different from natural infections [44]. Furthermore, the feasibility of developing a pregnancy malaria vaccine may depend on identifying structurally or functionally conserved epitopes that will not vary under vaccine pressure.

Because it is not yet known with certainty which regions of VAR2CSA bind to CSA, a reductionist approach has been taken to identify VAR2CSA domains that can induce adhesion blocking antibody responses. Recent work suggests that DBL4 and DBL5 recombinant proteins can induce inhibitory antibodies [31,33,34], sometimes of significant breadth [34], but inhibitory responses have been inconsistent between different antigen preparations. In addition, a full set of VAR2CSA domains has only been analysed from the *Baculovirus *system or by DNA vaccination, and differences have already been observed between different expression platforms [26,29,32]. This is an important consideration because the DBL domain has a highly complex protein fold with multiple disulfide bonds [45] and some expression systems may be more commercially scalable than others.

This study reports on the immunogenicity of single domain recombinant proteins produced in *P. pastoris*, which has also been employed to produce functionally active DBL domains from erythrocyte invasion ligands [45-47]. Antibody cross-reactivity was characterized on a panel of five heterologous CSA-binding parasites from different regions of the world. VAR2CSA recombinant proteins differed significantly in immunogenicity. DBL1 and DBL4 domains elicited very weak or no antibody responses against the homologous CSA-binding parasite, while the DBL6 domain elicited a strong antibody response to the homologous parasite variant, but did not cross-react against heterologous CSA-binding lines, possibly due to the greater polymorphism of this domain [18]. The DBL5 and especially the DBL3 domains were exceptional in that a single immunogen induced cross-reactive antibodies to diverse CSA-binding parasite lines in both rat and rabbit immunizations. Intriguingly, DBL3 and DBL5 are also the major targets of maternal antibodies cloned from pregnant women [48], indicating these domains are highly immunogenic in natural infections. Similar to vaccine antibodies, maternal monoclonal antibodies against DBL3 and DBL5 have only weak adhesion blocking activity [49], but recognize epitopes that are at least partially shared between different VAR2CSA alleles. While adhesion blocking antibodies are thought to make a major contribution to protection [6,7], cytophillic antibodies are the predominant isotype in pregnant women [50] and may also have a role in both protection [10] and monocyte-driven inflammatory complications of placental infections. It will be interesting to learn more about the specificity of vaccine induced DBL3/DBL5 antibodies compared to naturally acquired antibodies, and if these responses could be harnessed for vaccine development by accelerating phagocytic clearance of placental infected erythrocytes in primigravid women before severe inflammation develops.

In the full series of different DBL domains, inhibitory antibody responses were only observed in a single rat immunized with a 7G8-DBL4 recombinant protein. Altogether, five of the six DBL domains from 7G8-VAR2CSA were tested, but the DBL2 domain could not be produced and it was possible not evaluate whether it has potential to elicit adhesion blocking antibodies. The FCR3-DBL4 domain that has worked for others was also tested [31], but did not elicit inhibitory antibodies. However, but the DBL2 domain could not be produced and it was not possible to evaluate whether the two protein expression systems could not be directly compared. It has recently been reported that a full-length VAR2CSA extracellular domain (DBL1-6) induced potent adhesion blocking antibodies and was even more effective than the isolated DBL4 domain against the homologous parasite [24]. Thus, anti-VAR2CSA antibodies can block CSA-binding, but a challenge will be to focus antibodies on inhibitory epitopes in larger VAR2CSA vaccine immunogens or to define smaller immunogens that can consistently induce a broad inhibitory antibody response.

## Conclusions

This study assessed the immunogenicity of single domain VAR2CSA constructs produced in *P. pastoris*. Overall, single domain constructs were poor immunogens for adhesion blocking antibody responses, but DBL3 and DBL5 recombinant proteins induced cross-reactive antibodies to diverse CSA-binding parasite lines. Although VAR2CSA is unusually conserved for the *var *gene family, it is relatively polymorphic compared to most parasite proteins. The finding that VAR2CSA displays widely strain-transcendent antibody epitopes may have application for pregnancy malaria vaccine development.

## Abbreviations

CSA: Chondroitin sulfate A; DBL: Duffy binding-like; IE: infected erythrocyte; MFI: mean of fluorescence intensity

## Competing interests

The authors declare that they have no competing interests.

## Authors' contributions

MA and JDS conceived and designed the experiments; MA, MMC, and MJH performed the experiments; MA, MMC, MJH, JDS analysed the data; MA and JDS wrote the manuscript. All authors read and approved the final manuscript.
